# Supplementary material for: A Likelihood Approach for Real-Time Calibration of Stochastic Compartmental Epidemic Models
Source: PLoS Comput Biol. 2017 Jan 17;13(1):e1005257. doi: 10.1371/journal.pcbi.1005257 (PMC5240920; doi:10.1371/journal.pcbi.1005257)
Supplement: S1 File — (TAR.GZ) [file pcbi.1005257.s014.tar.gz › HSPH_Online-SI-Revision/Additions/PF09/n100-extreme-SEITR/n100-extreme-SEITR_PF2_table4.pdf]

1	newly 3 weeks spec	1057.	4069.55	{3985., 4138.}	285.009	0.585423	newly 3 weeks cumul	5452.	13 957.1	{13 849., 14 077.}	155.999	0.408232	newly 5 weeks spec	382.	2939.02	{2871., 3012.}	669.377	0.886067	newly 5 weeks cumul	6457.	20 369.3	{20 234., 20 519.}	215.461	0.498939	newly 47 weeks cumul	7014.	33 503.1	{33 262., 33 679.}	377.66	0.679113
2	newly 3 weeks spec	909.	4434.13	{4358., 4508.}	387.803	0.688208	newly 3 weeks cumul	5060.	17 355.1	{17 246., 17 458.}	242.986	0.535271	newly 5 weeks spec	300.	2591.53	{2532., 2656.}	763.843	0.936349	newly 5 weeks cumul	5879.	23 353.6	{23 241., 23 463.}	297.238	0.599048	newly 54 weeks cumul	6291.	31 215.2	{31 109., 31 336.}	396.189	0.695645
3	newly 3 weeks spec	1244.	4738.16	{4646., 4825.}	280.881	0.580747	newly 3 weeks cumul	6664.	18 342.1	{18 234., 18 476.}	175.241	0.439708	newly 5 weeks spec	463.	2798.97	{2736., 2859.}	504.529	0.781346	newly 5 weeks cumul	7872.	24 801.4	{24 684., 24 915.}	215.059	0.498389	newly 56 weeks cumul	8546.	33 381.7	{33 256., 33 507.}	290.612	0.591744
4	newly 3 weeks spec	1162.	3810.73	{3723., 3886.}	227.946	0.515738	newly 3 weeks cumul	6474.	15 083.9	{14 917., 15 235.}	132.992	0.367329	newly 5 weeks spec	412.	2202.44	{2140., 2258.}	434.573	0.72791	newly 5 weeks cumul	7613.	20 181.	{19 988., 20 318.}	165.087	0.42338	newly 54 weeks cumul	8186.	26 778.5	{26 576., 26 903.}	227.125	0.514709
5	newly 3 weeks spec	334.	4882.06	{4808., 4965.}	1361.69	1.16482	newly 3 weeks cumul	2304.	18 900.8	{18 787., 19 005.}	720.347	0.913993	newly 5 weeks spec	78.	2879.76	{2809., 2958.}	3592.	1.56718	newly 5 weeks cumul	2553.	25 556.2	{25 436., 25 663.}	901.027	1.00044	newly 56 weeks cumul	2676.	34 372.6	{34 245., 34 508.}	1184.48	1.10872
6	newly 3 weeks spec	210.	4207.45	{4122., 4287.}	1903.55	1.30174	newly 3 weeks cumul	1688.	16 532.7	{16 409., 16 637.}	879.425	0.990964	newly 5 weeks spec	40.	2438.23	{2382., 2500.}	5995.58	1.78494	newly 5 weeks cumul	1825.	22 175.9	{22 044., 22 288.}	1115.12	1.08462	newly 55 weeks cumul	1849.	29 538.2	{29 410., 29 660.}	1497.52	1.20345
7	newly 3 weeks spec	1990.	4153.53	{4064., 4234.}	108.72	0.319511	newly 3 weeks cumul	8888.	14 388.6	{14 269., 14 490.}	61.8883	0.209208	newly 5 weeks spec	982.	2959.54	{2886., 3038.}	201.379	0.479031	newly 5 weeks cumul	11 272.	20 861.9	{20 720., 20 966.}	85.0775	0.267348	newly 47 weeks cumul	13 516.	33 808.3	{33 562., 34 038.}	150.135	0.398168
8	newly 3 weeks spec	1262.	4116.97	{4035., 4187.}	226.226	0.51347	newly 3 weeks cumul	6258.	16 139.7	{16 021., 16 251.}	157.906	0.411455	newly 5 weeks spec	510.	2407.05	{2348., 2465.}	371.971	0.673832	newly 5 weeks cumul	7632.	21 698.1	{21 594., 21 803.}	184.304	0.45378	newly 54 weeks cumul	8594.	28 979.3	{28 873., 29 062.}	237.204	0.527891
9	newly 3 weeks spec	351.	5778.71	{5678., 5866.}	1546.36	1.21649	newly 3 weeks cumul	2373.	21 899.	{21 774., 22 028.}	822.841	0.965122	newly 5 weeks spec	96.	3471.	{3411., 3535.}	3515.63	1.55813	newly 5 weeks cumul	2626.	29 877.1	{29 736., 30 019.}	1037.74	1.05604	newly 56 weeks cumul	2718.	40 634.5	{40 524., 40 753.}	1395.01	1.17464
10	newly 3 weeks spec	2417.	4020.06	{3937., 4093.}	66.3244	0.220907	newly 3 weeks cumul	10 500.	15 385.6	{15 250., 15 508.}	46.5296	0.165916	newly 5 weeks spec	1167.	2434.98	{2369., 2506.}	108.653	0.319328	newly 5 weeks cumul	13 414.	20 955.	{20 785., 21 071.}	56.2172	0.193723	newly 52 weeks cumul	16 196.	28 834.6	{28 496., 29 042.}	78.035	0.250495
11	newly 3 weeks spec	2013.	5950.31	{5850., 6033.}	195.594	0.47066	newly 3 weeks cumul	9231.	19 853.1	{19 718., 19 985.}	115.07	0.332574	newly 5 weeks spec	1003.	4360.32	{4276., 4467.}	334.728	0.638161	newly 5 weeks cumul	11 702.	29 378.	{29 177., 29 535.}	151.051	0.399758	newly 51 weeks cumul	13 920.	47 964.9	{47 750., 48 165.}	244.576	0.537282
12	newly 3 weeks spec	1233.	3869.28	{3774., 3951.}	213.81	0.496594	newly 3 weeks cumul	6317.	14 974.8	{14 852., 15 077.}	137.056	0.374843	newly 5 weeks spec	542.	2307.14	{2247., 2374.}	325.672	0.628964	newly 5 weeks cumul	7681.	20 275.1	{20 174., 20 384.}	163.964	0.421541	newly 52 weeks cumul	8581.	27 552.5	{27 433., 27 669.}	221.087	0.50662
13	newly 3 weeks spec	1573.	4654.73	{4578., 4727.}	195.914	0.471129	newly 3 weeks cumul	7548.	17 620.1	{17 515., 17 729.}	133.44	0.368171	newly 5 weeks spec	706.	2864.89	{2804., 2921.}	305.792	0.608238	newly 5 weeks cumul	9330.	24 161.5	{24 055., 24 268.}	158.966	0.41324	newly 53 weeks cumul	10 733.	33 485.1	{33 355., 33 633.}	211.983	0.494128
14	newly 3 weeks spec	2628.	4515.31	{4423., 4590.}	71.8154	0.235016	newly 3 weeks cumul	11 099.	14 527.2	{14 427., 14 626.}	30.8878	0.116892	newly 5 weeks spec	1313.	3620.73	{3532., 3696.}	175.76	0.440456	newly 5 weeks cumul	14 282.	22 226.3	{22 074., 22 394.}	55.6246	0.192073	newly 46 weeks cumul	17 443.	42 007.9	{41 685., 42 296.}	140.83	0.381702
15	newly 3 weeks spec	1421.	4809.06	{4707., 4887.}	238.428	0.52941	newly 3 weeks cumul	6948.	14 884.6	{14 727., 15 006.}	114.229	0.330861	newly 5 weeks spec	621.	4090.08	{3999., 4180.}	558.628	0.81857	newly 5 weeks cumul	8459.	23 470.8	{23 276., 23 663.}	177.465	0.443196	newly 47 weeks cumul	9505.	47 840.6	{47 540., 48 046.}	403.32	0.701838
16	newly 3 weeks spec	1685.	4244.96	{4160., 4339.}	151.926	0.401216	newly 3 weeks cumul	8356.	15 367.1	{15 239., 15 475.}	83.9053	0.264587	newly 5 weeks spec	644.	2775.13	{2702., 2845.}	330.921	0.634311	newly 5 weeks cumul	10 017.	21 597.9	{21 466., 21 747.}	115.613	0.333669	newly 50 weeks cumul	11 020.	31 474.2	{31 214., 31 684.}	185.61	0.455766
17	newly 3 weeks spec	244.	4917.4	{4824., 4989.}	1915.33	1.30431	newly 3 weeks cumul	2213.	19 084.7	{18 968., 19 217.}	762.392	0.935699	newly 5 weeks spec	41.	2883.82	{2811., 2962.}	6933.71	1.84711	newly 5 weeks cumul	2358.	25 746.1	{25 617., 25 855.}	991.862	1.03817	newly 56 weeks cumul	2381.	34 481.6	{34 375., 34 598.}	1348.2	1.16083
18	newly 3 weeks spec	946.	4916.71	{4854., 4992.}	419.737	0.715754	newly 3 weeks cumul	4981.	18 992.1	{18 894., 19 094.}	281.291	0.581252	newly 5 weeks spec	314.	2920.42	{2853., 2986.}	830.07	0.96845	newly 5 weeks cumul	5835.	25 727.7	{25 617., 25 832.}	340.921	0.644358	newly 55 weeks cumul	6316.	34 703.	{34 612., 34 799.}	449.445	0.739923
19	newly 3 weeks spec	2246.	5589.48	{5488., 5660.}	148.864	0.395929	newly 3 weeks cumul	9933.	18 810.6	{18 644., 18 935.}	89.3743	0.277315	newly 5 weeks spec	1136.	4039.02	{3955., 4118.}	255.548	0.55084	newly 5 weeks cumul	12 584.	27 657.7	{27 505., 27 803.}	119.785	0.341994	newly 50 weeks cumul	14 784.	44 875.8	{44 600., 45 132.}	203.543	0.482216
20	newly 3 weeks spec	688.	4268.38	{4188., 4364.}	520.404	0.792621	newly 3 weeks cumul	3837.	16 758.7	{16 635., 16 859.}	336.765	0.640242	newly 5 weeks spec	219.	2478.26	{2416., 2549.}	1031.63	1.05362	newly 5 weeks cumul	4426.	22 500.5	{22 384., 22 613.}	408.371	0.706178	newly 55 weeks cumul	4693.	29 976.1	{29 844., 30 087.}	538.742	0.805323
21	newly 3 weeks spec	566.	5066.55	{4969., 5155.}	795.15	0.951852	newly 3 weeks cumul	3401.	19 590.	{19 494., 19 703.}	476.007	0.760423	newly 5 weeks spec	156.	2987.1	{2917., 3052.}	1814.81	1.28205	newly 5 weeks cumul	3828.	26 492.9	{26 381., 26 608.}	592.081	0.840154	newly 56 weeks cumul	3988.	35 634.1	{35 514., 35 751.}	793.534	0.95111
22	newly 3 weeks spec	1150.	3413.37	{3335., 3502.}	196.815	0.472418	newly 3 weeks cumul	5834.	13 506.4	{13 360., 13 647.}	131.511	0.364558	newly 5 weeks spec	400.	1991.26	{1928., 2050.}	397.815	0.696953	newly 5 weeks cumul	6931.	18 129.4	{17 986., 18 241.}	161.57	0.41758	newly 52 weeks cumul	7573.	24 213.	{24 059., 24 362.}	219.727	0.504775
23	newly 3 weeks spec	361.	3141.87	{3073., 3215.}	770.324	0.939613	newly 3 weeks cumul	2348.	12 523.2	{12 433., 12 611.}	433.354	0.727009	newly 5 weeks spec	79.	1819.82	{1765., 1875.}	2203.57	1.36229	newly 5 weeks cumul	2588.	16 745.2	{16 669., 16 833.}	547.034	0.810923	newly 53 weeks cumul	2670.	22 242.3	{22 150., 22 331.}	733.046	0.920667
24	newly 3 weeks spec	617.	5389.62	{5301., 5472.}	773.52	0.941244	newly 3 weeks cumul	3939.	20 643.4	{20 506., 20 772.}	424.078	0.719391	newly 5 weeks spec	171.	3208.8	{3137., 3281.}	1776.49	1.27328	newly 5 weeks cumul	4404.	28 050.9	{27 906., 28 176.}	536.942	0.804097	newly 55 weeks cumul	4602.	37 946.1	{37 819., 38 067.}	724.557	0.91622
25	newly 3 weeks spec	1330.	4836.89	{4752., 4935.}	263.676	0.560672	newly 3 weeks cumul	7007.	17 388.4	{17 268., 17 519.}	148.158	0.394721	newly 5 weeks spec	443.	3144.47	{3061., 3219.}	609.813	0.851065	newly 5 weeks cumul	8227.	24 467.8	{24 340., 24 603.}	197.408	0.473348	newly 51 weeks cumul	8870.	35 326.1	{35 027., 35 596.}	298.264	0.600164
26	newly 3 weeks spec	920.	3602.84	{3526., 3692.}	291.613	0.592792	newly 3 weeks cumul	5136.	13 407.9	{13 313., 13 504.}	161.057	0.416728	newly 5 weeks spec	345.	2277.89	{2213., 2353.}	560.258	0.81958	newly 5 weeks cumul	6057.	18 565.4	{18 422., 18 682.}	206.511	0.486438	newly 49 weeks cumul	6590.	26 388.3	{26 101., 26 595.}	300.43	0.602513
27	newly 3 weeks spec	2118.	3833.56	{3749., 3927.}	80.9991	0.2576	newly 3 weeks cumul	9250.	14 266.1	{14 143., 14 390.}	54.2277	0.188153	newly 5 weeks spec	1079.	2418.04	{2350., 2493.}	124.1	0.350317	newly 5 weeks cumul	11 803.	19 730.	{19 553., 19 894.}	67.1606	0.223125	newly 49 weeks cumul	14 480.	27 889.4	{27 591., 28 215.}	92.6066	0.284656
28	newly 3 weeks spec	196.	4430.03	{4341., 4518.}	2160.22	1.3541	newly 3 weeks cumul	1632.	17 293.6	{17 182., 17 391.}	959.656	1.02516	newly 5 weeks spec	32.	2596.22	{2525., 2666.}	8013.19	1.90911	newly 5 weeks cumul	1750.	23 300.7	{23 160., 23 407.}	1231.47	1.12433	newly 56 weeks cumul	1781.	31 192.4	{31 078., 31 320.}	1651.4	1.24338
29	newly 3 weeks spec	1912.	4116.49	{4034., 4188.}	115.298	0.332995	newly 3 weeks cumul	9043.	16 055.3	{15 936., 16 162.}	77.5444	0.249301	newly 5 weeks spec	849.	2422.74	{2368., 2493.}	185.364	0.455322	newly 5 weeks cumul	11 162.	21 627.6	{21 536., 21 728.}	93.7611	0.287264	newly 53 weeks cumul	12 786.	29 058.	{28 934., 29 147.}	127.264	0.356529
30	newly 3 weeks spec	1221.	5435.94	{5342., 5520.}	345.204	0.648526	newly 3 weeks cumul	6812.	20 875.4	{20 724., 20 978.}	206.45	0.486354	newly 5 weeks spec	428.	3230.56	{3159., 3306.}	654.804	0.877762	newly 5 weeks cumul	7969.	28 325.7	{28 221., 28 410.}	255.448	0.550775	newly 56 weeks cumul	8592.	38 222.2	{38 107., 38 322.}	344.858	0.648221
31	newly 3 weeks spec	1586.	3116.12	{3035., 3206.}	96.4767	0.293221	newly 3 weeks cumul	7252.	11 559.4	{11 459., 11 656.}	59.3956	0.202467	newly 5 weeks spec	689.	2031.12	{1979., 2083.}	194.792	0.469424	newly 5 weeks cumul	9024.	16 121.5	{16 015., 16 214.}	78.6512	0.252	newly 46 weeks cumul	10 290.	23 760.5	{23 569., 23 904.}	130.909	0.363434
32	newly 3 weeks spec	2184.	3235.3	{3162., 3301.}	48.1364	0.170596	newly 3 weeks cumul	9583.	12 812.3	{12 713., 12 901.}	33.6977	0.126115	newly 5 weeks spec	1094.	1874.29	{1814., 1927.}	71.3245	0.233671	newly 5 weeks cumul	12 124.	17 163.3	{17 038., 17 303.}	41.5643	0.150946	newly 52 weeks cumul	14 626.	22 873.5	{22 736., 23 023.}	56.3896	0.194203
33	newly 3 weeks spec	643.	3976.81	{3890., 4068.}	518.477	0.791263	newly 3 weeks cumul	3804.	15 708.7	{15 605., 15 817.}	312.953	0.615894	newly 5 weeks spec	197.	2290.96	{2228., 2359.}	1062.92	1.06545	newly 5 weeks cumul	4382.	21 024.5	{20 893., 21 132.}	379.793	0.68105	newly 56 weeks cumul	4693.	27 895.3	{27 786., 27 993.}	494.401	0.774078
34	newly 3 weeks spec	883.	3010.85	{2942., 3080.}	240.98	0.53266	newly 3 weeks cumul	4731.	11 996.7	{11 896., 12 097.}	153.576	0.4041	newly 5 weeks spec	278.	1749.44	{1693., 1804.}	529.295	0.798715	newly 5 weeks cumul	5501.	16 056.5	{15 970., 16 139.}	191.883	0.465205	newly 52 weeks cumul	5915.	21 362.2	{21 259., 21 467.}	261.153	0.557688
35	newly 3 weeks spec	239.	4953.57	{4869., 5031.}	1972.62	1.31648	newly 3 weeks cumul	1921.	19 183.2	{19 042., 19 306.}	898.606	0.999388	newly 5 weeks spec	50.	2932.97	{2873., 2996.}	5765.94	1.76827	newly 5 weeks cumul	2064.	25 952.1	{25 797., 26 068.}	1157.37	1.09946	newly 56 weeks cumul	2096.	34 900.3	{34 779., 35 012.}	1565.09	1.22144
36	newly 3 weeks spec	2432.	4356.2	{4280., 4424.}	79.1201	0.253106	newly 3 weeks cumul	10 891.	16 871.7	{16 733., 16 978.}	54.9145	0.190086	newly 5 weeks spec	1211.	2579.87	{2531., 2640.}	113.036	0.328389	newly 5 weeks cumul	13 782.	22 808.3	{22 700., 22 913.}	65.4932	0.218777	newly 52 weeks cumul	16 479.	30 789.3	{30 641., 30 916.}	86.8397	0.271466
37	newly 3 weeks spec	1023.	5839.97	{5741., 5932.}	470.867	0.756503	newly 3 weeks cumul	5532.	21 625.3	{21 499., 21 744.}	290.913	0.592076	newly 5 weeks spec	383.	3624.87	{3549., 3701.}	846.441	0.976034	newly 5 weeks cumul	6497.	29 876.3	{29 742., 30 003.}	359.848	0.662612	newly 53 weeks cumul	7036.	41 628.1	{41 474., 41 774.}	491.645	0.772059
38	newly 3 weeks spec	915.	4114.25	{4026., 4205.}	349.645	0.652812	newly 3 weeks cumul	5124.	15 064.9	{14 955., 15 174.}	194.007	0.46835	newly 5 weeks spec	323.	2630.	{2543., 2704.}	714.241	0.910625	newly 5 weeks cumul	5962.	20 992.8	{20 823., 21 145.}	252.111	0.546672	newly 50 weeks cumul	6391.	30 028.5	{29 756., 30 297.}	369.856	0.671948
39	newly 3 weeks spec	1339.	3812.17	{3741., 3884.}	184.703	0.454341	newly 3 weeks cumul	6703.	14 745.8	{14 654., 14 860.}	119.988	0.342394	newly 5 weeks spec	464.	2295.24	{2240., 2351.}	394.664	0.694224	newly 5 weeks cumul	7906.	20 004.5	{19 906., 20 097.}	153.029	0.403167	newly 52 weeks cumul	8666.	27 272.4	{27 145., 27 387.}	214.705	0.497902
40	newly 3 weeks spec	1822.	3168.24	{3096., 3234.}	73.888	0.240207	newly 3 weeks cumul	8114.	12 565.2	{12 479., 12 652.}	54.8578	0.189927	newly 5 weeks spec	874.	1841.21	{1785., 1886.}	110.665	0.323488	newly 5 weeks cumul	10 273.	16 840.5	{16 730., 16 926.}	63.9295	0.214653	newly 53 weeks cumul	12 291.	22 454.8	{22 339., 22 554.}	82.6934	0.26172
41	newly 3 weeks spec	362.	5470.57	{5371., 5564.}	1411.21	1.17929	newly 3 weeks cumul	2774.	20 853.4	{20 734., 20 965.}	651.746	0.876067	newly 5 weeks spec	74.	3280.03	{3207., 3342.}	4332.47	1.64659	newly 5 weeks cumul	2996.	28 396.4	{28 278., 28 518.}	847.81	0.976719	newly 56 weeks cumul	3059.	38 534.4	{38 435., 38 642.}	1159.7	1.10027
42	newly 3 weeks spec	2612.	3690.23	{3605., 3773.}	41.2799	0.150009	newly 3 weeks cumul	10 679.	13 415.	{13 290., 13 522.}	25.6207	0.0990524	newly 5 weeks spec	1374.	2433.77	{2360., 2507.}	77.1303	0.248185	newly 5 weeks cumul	13 825.	18 865.7	{18 730., 18 984.}	36.4607	0.135002	newly 47 weeks cumul	17 141.	28 293.9	{28 093., 28 477.}	65.0653	0.21765
43	newly 3 weeks spec	2253.	3829.69	{3750., 3902.}	69.9818	0.23035	newly 3 weeks cumul	10 165.	14 817.7	{14 706., 14 941.}	45.772	0.163666	newly 5 weeks spec	1018.	2296.8	{2234., 2357.}	125.619	0.353283	newly 5 weeks cumul	12 779.	20 089.1	{19 970., 20 204.}	57.2041	0.196459	newly 52 weeks cumul	15 131.	27 374.8	{27 196., 27 539.}	80.9183	0.257477
44	newly 3 weeks spec	1384.	3801.45	{3725., 3864.}	174.671	0.438766	newly 3 weeks cumul	6755.	14 934.4	{14 815., 15 057.}	121.087	0.344554	newly 5 weeks spec	626.	2224.01	{2160., 2304.}	255.273	0.550437	newly 5 weeks cumul	8295.	20 067.2	{19 944., 20 172.}	141.92	0.383667	newly 52 weeks cumul	9359.	26 842.5	{26 742., 26 945.}	186.809	0.457591
45	newly 3 weeks spec	1196.	4052.7	{3973., 4127.}	238.855	0.529963	newly 3 weeks cumul	6409.	15 907.	{15 805., 16 012.}	148.197	0.394792	newly 5 weeks spec	536.	2361.9	{2296., 2423.}	340.653	0.644011	newly 5 weeks cumul	7757.	21 361.6	{21 251., 21 477.}	175.384	0.439936	newly 54 weeks cumul	8640.	28 516.1	{28 412., 28 615.}	230.048	0.518575
46	newly 3 weeks spec	1149.	4693.89	{4598., 4790.}	308.52	0.611159	newly 3 weeks cumul	6165.	18 230.4	{18 119., 18 344.}	195.709	0.470859	newly 5 weeks spec	377.	2769.07	{2709., 2823.}	634.501	0.865934	newly 5 weeks cumul	7203.	24 633.3	{24 530., 24 734.}	241.986	0.534006	newly 56 weeks cumul	7768.	33 131.	{33 030., 33 237.}	326.507	0.629924
47	newly 3 weeks spec	1358.	5078.95	{4989., 5192.}	274.002	0.572824	newly 3 weeks cumul	7212.	18 085.	{17 965., 18 202.}	150.763	0.399257	newly 5 weeks spec	550.	3362.6	{3273., 3437.}	511.382	0.78624	newly 5 weeks cumul	8640.	25 622.6	{25 465., 25 762.}	196.558	0.472106	newly 51 weeks cumul	9536.	37 627.6	{37 413., 37 865.}	294.585	0.596134
48	newly 3 weeks spec	874.	4367.03	{4287., 4437.}	399.66	0.698625	newly 3 weeks cumul	5250.	17 107.2	{17 015., 17 206.}	225.851	0.513014	newly 5 weeks spec	248.	2555.63	{2478., 2619.}	930.496	1.01295	newly 5 weeks cumul	5990.	23 024.	{22 916., 23 132.}	284.373	0.58475	newly 54 weeks cumul	6323.	30 782.3	{30 695., 30 875.}	386.83	0.687376
49	newly 3 weeks spec	879.	2716.05	{2650., 2781.}	208.993	0.489871	newly 3 weeks cumul	4780.	10 894.2	{10 809., 11 001.}	127.911	0.35775	newly 5 weeks spec	321.	1562.25	{1510., 1613.}	386.682	0.687137	newly 5 weeks cumul	5641.	14 517.2	{14 381., 14 637.}	157.352	0.410514	newly 52 weeks cumul	6148.	19 212.7	{19 082., 19 328.}	212.504	0.494845
50	newly 3 weeks spec	1513.	3382.08	{3304., 3473.}	123.535	0.349267	newly 3 weeks cumul	7746.	13 459.8	{13 351., 13 560.}	73.7647	0.239953	newly 5 weeks spec	636.	1947.39	{1871., 2002.}	206.193	0.485865	newly 5 weeks cumul	9438.	17 998.5	{17 890., 18 096.}	90.7021	0.280349	newly 53 weeks cumul	10 556.	23 932.9	{23 799., 24 075.}	126.723	0.355489
